# Job Quality and Job Separation of Direct Care Workers in England

**DOI:** 10.1093/geroni/igad009

**Published:** 2023-01-31

**Authors:** Florin Vadean, Eirini-Christina Saloniki

**Affiliations:** Personal Social Services Research Unit (PSSRU), University of Kent, Canterbury, UK; Global Labor Organization (GLO), Essen, Germany; Department of Applied Health Research, University College London, London, UK; National Institute for Health and Care Research (NIHR) Applied Research Collaboration North Thames, London, UK

**Keywords:** Long-term care, Panel data, Staff turnover, Workforce

## Abstract

**Background and Objectives:**

Most job leavers in the long-term care (LTC) sector in England do not leave the sector, but rather move to other LTC employers. Nevertheless, the high “churn” can have a negative impact on continuity and quality of care, care providers’ recruitment and training costs, and the remaining staff workload and motivation. This study aimed to provide quantitative evidence on the drivers of direct care workers’ job separation in England, with a focus on job quality.

**Research Design and Methods:**

We used yearly data (2016–19) from the Adult Social Care Workforce Data Set, the leading source of LTC workforce data in England, including information on both LTC workers and employers. The analysis considered panel data econometric methods that accounted for unobserved heterogeneity at worker and employer levels.

**Results:**

After controlling for observed individual, organizational, and local market characteristics as well as unobserved worker and employer heterogeneity, we found that everything else being equal, wages and employment conditions (i.e., full-time contracts and contracts with guaranteed working hours) significantly reduce job separation. For example, a 10% wage increase from the sample mean would reduce the job separation rate by about 3 percentage points. This wage effect was more than halved (i.e., downward biased) when not accounting for unobserved effects.

**Discussion and Implications:**

The persistent high staff turnover in LTC in England highlights the need for finding practical solutions faced by care providers and policy-makers. Our findings showed that improving pay and employment conditions can be the way forward while methodologically stressing the importance of accounting for unobserved variable bias.

**Translational Significance:** The persistently high staff turnover rates in the long-term care (LTC) sector in England and elsewhere have a negative impact on providers’ costs as well as the quality of care. The findings of this study show that direct care staff turnover can be substantially reduced by increasing pay and improving employment conditions. To achieve this, the LTC sector in England would need additional government funding. The potential gains are, however, important, including a better motivated workforce, reduction in care providers’ recruitment and induction costs, as well as an improvement in the continuity and quality of care for the large population receiving LTC.

## Background and Objectives

At over 30% in 2019/20, the long-term care (LTC) staff turnover rate in England is perceived to be relatively high (Skills for Care, 2020b). The turnover rates are higher in the independent sector (34%) than the public sector (13%). With respect to job roles, turnover rates are highest (38%) among direct care workers who support people with all aspects of their daily living (e.g., personal care, social and physical activities, and meals). Although the majority of job leavers (about 66%) do not leave the sector, but rather move to other LTC employers, there are concerns that the high turnover rate has a negative impact on the continuity and quality of care, care providers’ recruitment and training costs, and the remaining direct care workers’ workload and motivation (Allan and Vadean, 2021; Buchan, 2010; Castle and Engberg, 2005; Eastwood, 2017; Netten et al., 2005, 2007; Seavey, 2004; Skills for Care, 2020b).

While most care providers and government agencies are favoring the improvement of staff retention (Health Education England, 2017; National Audit Office, 2018; The Health Foundation et al., 2018), LTC staff turnover in England increased by about 10 percentage points (ppt) during the last decade (Skills for Care, 2020b). The most common factors assumed to be related to low retention in LTC are low pay levels (often at minimum wage), lack of status (as care work is not recognized as a profession), limited opportunities for career progression, and employment without guaranteed hours (i.e., so-called zero-hours contracts; Health Education England, 2017; Moriarty et al., 2018; National Audit Office, 2018).

Despite the importance of improving staff retention in LTC, there is no hard evidence on what factors drive the retention of direct care workers in England. The aim of this study was to address this research gap, with a focus on job quality (e.g., wages and guaranteed working hours). The study also extended existing (mainly U.S.) literature on the determinants of LTC staff turnover by utilizing rich panel data for several years and controlling for unobserved heterogeneity at both worker and employer levels. We used yearly data for 2016–19 from the Adult Social Care Workforce Data Set (ASC-WDS), the main source of LTC workforce information in England.

### Factors Affecting Turnover

There is a growing literature on the determinants of LTC workforce job separation and turnover—see Turnpenny and Hussein (2020) for a review—revealing the importance of various factors associated with worker, job, employer, and local market characteristics.

At a local level, lower unemployment and higher competition have been found to be positively associated with a higher turnover of direct care workers (Castle, 2008; Donoghue, 2010; Vadean and Saloniki, 2020), while wages of alternative job opportunities in the same locality can further influence this relationship. Failure to adequately control for local labor market characteristics (including wages) can potentially bias the estimated effects (Manning, 2003).

At the organizational level, care establishment size, for-profit ownership, home care provision, lower staffing levels, and a higher share of direct care workers on contracts without guaranteed hours have been found to be associated with higher turnover rates (Castle, 2008; Castle and Engberg, 2006; Hussein et al., 2016; Vadean and Saloniki, 2020). The management style also mattered. For example, practices that gave direct care workers higher job autonomy and/or empowered them to participate in service users’ care planning reduced direct care workers’ turnover (Donoghue and Castle, 2009).

In terms of job-related characteristics, higher turnover has previously been found to be related to part-time employment, tenure, work overload, work stress, low levels of support from supervisors and coworkers, as well as satisfaction with training and rewards (Castle et al., 2007; Gao et al., 2014; Gaudenz et al., 2019; Ha et al., 2014; Karantzas et al., 2012; Morris, 2009; Park et al., 2017; Rosen et al., 2011; Yeatts et al., 2010). There are arguments both for including and excluding job tenure from models of job separation (Manning, 2003). On the one hand, paying higher wages is expected to reduce separations (and increase tenure), which would suggest that tenure should not be included. On the other hand, if there are seniority wage scales, the exclusion of tenure can lead to biased estimates. In this study, we followed Frijters et al. (2007), Hirsch and Jahn (2015), and Vick (2017), and controlled for tenure as we believe that helps better deal with unobserved heterogeneity, which is an important issue in this context.

Finally, the influence of pay on turnover in LTC has been mixed and is based mainly on cross-section studies from the United States. For example, Rosen et al. (2011) found that hourly pay did not predict the intention of direct care workers to leave their job and argued that this may be explained by the fact that any pecuniary benefits (i.e., pay and rewards) may be offset by non-pecuniary and indirect costs associated with the status quo. On the other hand, using a sample of 507 home care workers from Maine, Morris (2009) found that job separation was negatively related to hourly wages and that a switch to another nursing care job was significantly associated with a wage increase. Similarly, Howes (2005) found that the doubling of wages of independent homecare providers (i.e., personal assistants in the United Kingdom) in San Francisco County between 1996 and 2002 significantly increased their retention, while Rapp and Sicsic (2020) using pooled individual data from one of the largest U.S. household surveys identified a positive relationship between hourly wages and staying in the LTC sector.

It is important to note that unobserved heterogeneity has not been accounted for in the above studies, but has been found to bias wage effects toward zero in studies on other sectors (Manning, 2003). One of the few studies that took into account the endogeneity of wages in the LTC context is Powers and Powers (2010). Using data from 61 community-based care sites in Illinois and instrumental variable methods to identify the effect of wages on employer-level turnover rates, they found a negative effect and a significant downward bias when not controlling for unobserved factors. A study accounting for the endogeneity of wages in an individual-level analysis of decisions to move out of a direct care occupation is Baughman and Smith (2012). Using nationally representative data for the United States and the presence of a state Medicaid wage pass-through program as the main exogenous instrument for wages, they found only a modest effect of wages increasing the duration of employment in a direct care occupation.

Given the existing research findings, our hypothesis is that job quality (e.g., wages and guaranteed working hours) will significantly reduce job separations. We also expect estimation methods controlling for unobserved heterogeneity to be important in reducing unobserved variable bias. The data and estimation methods are described in the next section.

## Research Design and Methods

### Data and Measures

We used data from the ASC-WDS, an online data collection service managed by Skills for Care and a leading source of workforce information for the LTC sector in England. The information is rich at both establishment (e.g., type of service provided, sector, establishment size, count of employees and job roles, starters, leavers, vacancies, etc.) and worker levels (e.g., age, gender, nationality, qualifications, pay, working hours, job role, and job type). Public employers update their records on a mandatory basis in September each year, whereas independent employers submit data on a voluntary basis, but are incentivized to do so by having access to workforce development grants. All data in the ASC-WDS have been updated or confirmed to be so within the last 2 years. Moreover, about 80% of employers have updated their data in the past 6 months. Although the data set does not cover all independent sector establishments, it has a large enough sample to provide a solid basis for reliable workforce estimates at both national and local levels; for more information, see Skills for Care (2020a, 2020b).

Four cuts of the ASC-WDS were used: October 2016 to October 2019, matched at individual level, and with some variables from the provider data set. Skills for Care assigns to each establishment a unique and permanent ID and generates a unique and permanent ID for each worker based on national insurance number and date of birth. We excluded observations from all establishments with records not updated for more than 6 months and establishments that had unique IDs for less than 75% of their workers. We kept establishments providing either care home services (with or without nursing) or domiciliary care to adults (i.e., service users aged 18 and over). Statutory local authority, for-profit, and not-for-profit providers were all included.

We included in the sample employees aged between 16 and 64 in a direct care role, that is, care workers, senior care workers, and other care-providing roles (e.g., community support and outreach and activity workers). We excluded direct care workers without a unique ID as these could not be traced over time (7%), those who erroneously had multiple entries per year with the same establishment (1%), and direct care workers with two or more jobs in any year (6%). The last exclusion was mainly for domiciliary direct care workers. Over half of them are employed on contracts without guaranteed working hours and have no obligation to work a minimum number of hours per week. Therefore, some hold contracts with multiple agencies and it is usually not possible to correctly establish a job transition from one employer to another.

The job separation variable was defined as equal to “0” (i.e., stayer) if the employee was still with the same employer 1 year later (t+1), and equal to “1” (i.e., leaver) if either (a) the employee could be identified as working for another LTC employer in the sample at t+1; or (b) the employee left the sample, but their employer at time *t* was still in the sample at t+1. For a small number of cases for which information was missing at t+1 but available in a subsequent year, we used the information from the next available year to identify the job separation status. Employees for whom the job separation status could not be identified, because both they and their initial employer dropped from the sample in all subsequent years, were excluded from the analysis (14%). Our definition of job separation, therefore, refers to separations from the employer, and not necessarily a change in occupation or sector of employment. The final sample after excluding all observations with missing values for the relevant variables is an unbalanced panel consisting of 355,155 observations of 211,283 unique job spells in 8,312 care establishments.

### Sample Representativeness and Postsampling Weights

To determine the national representativeness of establishments in the analyzed sample, we compared its characteristics (i.e., sector, care home service type, care home capacity, overall quality rating, and regional distribution) with those of all adult LTC establishments regulated by the Care Quality Commission (CQC; see Supplementary Table A1).

We used raking to generate weights for each establishment (and year) using control totals obtained from the CQC care directory data, so that the weighted averages of the analyzed sample matched the average characteristics of all establishments in England. Following Valliant and Dever (2018), we compared the results of logistic regressions of job separation (a) without using weights, (b) with using weights, and (c) in which we included interactions between weights and the independent variables included in the main analysis. The adjusted *R*-squares from the three logistic regressions were very similar (i.e., 0.051, 0.048, and 0.052 for residential care; and 0.042, 0.040, and 0.045 for domiciliary care), indicating that unweighted regression analysis will give consistent estimates. Nonetheless, we used weights for computing the mean values presented in the descriptive statistics.

### Econometric Framework

For comparison with previous studies on the determinants of direct care workers’ intentions to leave and actual voluntary turnover (e.g., Castle et al., 2007; Morris, 2009; Rosen et al., 2011), we started the multivariate regression analysis with a pooled logistic regression. We also estimated a pooled probit and pooled linear probability model (LPM) to serve as a baseline for the models controlling for unobserved heterogeneity presented below. The Huber–White sandwich estimator clustered by job spell was used to obtain robust standard errors.

In the absence of good instruments, similar to the state Medicaid wage pass-through program used by Baughman and Smith (2012) to account for the endogeneity of wages, our strategy to account for unobserved variable bias was to make use of the longitudinal dimension of the data to control for time-invariant worker- and employer-unobserved heterogeneity. The preferred model was correlated random effects (CRE) probit. By including as covariates the averages over time of the time-variant variables, the estimated coefficients of the time-variant variables of the CRE probit model are Mundlak-type *within* effects. Fixed-effects (FE) LPM estimations were run for comparison, as they are easy to interpret. Although both yields were *within* estimates, CRE probit was preferred due to the binary nature of the dependent variable. Moreover, we preferred CRE probit over conditional FE logit, as it is a more flexible estimator (i.e., does not exclude groups for which the dependent variable does not change over time, like job spells that do not end into separation) and allows the identification of average partial effects (Wooldridge, 2010). The probability of a job separation is given by


$$\begin{array}{ll} & P\left(y_{ijt+1}=1|x_{ijt},c_{ij}\right)=\;\Phi \;\left(x_{ijt}\beta +c_{ij}\right)\\ & =\;\Phi \;\left(x_{ijt}\beta_{CREprobit}+{\bar{z}}_{ij}\xi_{CREprobit}+a_{ij}\right) \end{array}$$ (1)

where $y_{ijt+1}$ is a binary response equal to one if worker *i* separated from employer *j* between *t* and t+1 and zero if the job spell continued at t+1; $x_{ijt}$ is a set of explanatory variables at worker, employer, and local area level as described below; ${\bar{z}}_{ij}$is the average over time of the subset of time-varying variables included in$x_{ijt}$; and $a_{ij}$ is the part of the unobserved heterogeneity independent of the subset of time-varying variables in $x_{ijt}$ (Wooldridge, 2010). As the unit of observation is the job spell (i.e., unique worker–establishment combination), the estimator controls for (time-invariant) unobserved heterogeneity at both worker and employer levels.

Not controlling for unobserved heterogeneity can lead to either upward- or downward-biased results. As mentioned earlier, omitted variable bias has been found to bias wage effects on turnover or job separations toward zero (Baughman and Smith, 2012; Manning, 2003; Powers and Powers, 2010). For example, job stability might be the result of a good match, which could make job separation less likely. A direct care worker might have the right care ethic and motivation and/or the employer may give direct care workers the right type of support to deliver good-quality care. In this case, the good match could compensate for a lower wage (and employment conditions) and lead to a downward bias. Such unobservables would be time invariant (or change very little over time) and, thus be captured by ${\bar{z}}_{ij}$. Nonetheless, even if they change over time in a deterministic way, they would be captured by year-fixed effects.

The risk of omitted variable bias is also reduced by controlling for a large number of worker, job, employer, and local market characteristics. At the employer level, we included controls for sector (i.e., public, for-profit, and not-for-profit), user type (i.e., younger adults, older people, and mixed), establishment size, the direct care worker per service user ratio (as a proxy for workload), the vacancy rate in the previous year (as a proxy for difficulties with hiring sufficient direct care workers), as well as the independent care regulator rating of the management (i.e., the CQC rating on the Key Line of Enquiry “Well-led”). Moreover, we included the turnover rate in the previous year to capture any potential “chain” effects with respect to separations. Job-related characteristics at the worker level included job role, tenure (i.e., number of years with the current employer), training incidence, the log of hourly wage, full-time employment, and employment on contract without guaranteed working hours (i.e., zero-hours contract). At the local level, we controlled for the unemployment rate, the average wage for women, a measure of competition in the local LTC market, wealth (i.e., average house prices), as well as the LTC tariffs paid by local councils with LTC responsibilities, which could impact the revenue of LTC establishments and implicitly the pay and employment conditions offered to direct care workers.

## Results

### Descriptive Statistics

Job leavers were on average 3.6–3.9 years younger compared to stayers, living between 0.6 and 0.9 km further away from their place of work, were less likely to have a formal qualification, less likely to have received training if they worked in a care home (−3.7 ppt), and slightly more likely to have received training if they worked in domiciliary care (+1.3 ppt), had a shorter tenure with their employer (1.3 years shorter for domiciliary care and 2 years shorter for residential care), had slightly lower wages (−4 pence per hour for domiciliary care and −15 pence per hour for residential care), were more likely to be employed on a zero-hours contract, and less likely to be employed full time (Table 1).

**Table 1.** Descriptive Statistics—Direct Care Workers Aged 16–64 by Care Setting; Pooled Adult Social Care Workforce Data Set (ASC-WDS) Data (2016–18)

Variable	Residential Care				Domiciliary Care			
	Stayer	Leaver			Stayer	Leaver		
	Mean	Mean	Diff		Mean	Mean	Diff	
Age	41.252	37.355	3.897	***	42.808	39.184	3.624	***
Gender: female	0.864	0.845	0.019	***	0.868	0.877	−0.009	***
Nationality: British	0.828	0.809	0.018	***	0.855	0.847	0.008	***
Distance to work (km)	2.990	3.594	−0.604	***	6.183	7.058	−0.875	***
Qualification: yes	0.658	0.537	0.121	***	0.537	0.461	0.076	***
Training (any): yes	0.590	0.553	0.037	***	0.669	0.682	−0.013	***
Job tenure (years)	6.289	4.315	1.975	***	5.120	3.821	1.299	***
Job role: senior care worker	0.173	0.136	0.037	***	0.058	0.041	0.016	***
Job role: care worker	0.807	0.846	−0.040	***	0.879	0.907	−0.028	***
Job role: other care providing	0.021	0.018	0.003	***	0.064	0.052	0.012	***
Hourly wage (2015 £)	7.701	7.549	0.152	***	7.892	7.855	0.037	***
Zero-hours contract	0.025	0.063	−0.037	***	0.603	0.637	−0.034	***
Full time	0.591	0.570	0.021	***	0.489	0.472	0.017	***
Sector: statutory LA	0.032	0.020	0.012	***	0.036	0.029	0.007	***
Sector: for-profit	0.826	0.872	−0.046	***	0.819	0.864	−0.045	***
Sector: not-for-profit	0.142	0.108	0.034	***	0.145	0.107	0.038	***
Care type: care home with nursing	0.392	0.416	−0.024	***				
Care type: care home without nursing	0.608	0.584	0.024	***				
User type: old age	0.511	0.530	−0.019	***	0.080	0.071	0.009	***
User type: young adults	0.259	0.250	0.009	***	0.143	0.119	0.024	***
User type: mixed	0.230	0.220	0.010	***	0.777	0.810	−0.033	***
Staff size: micro/small (1–49 workers)	0.551	0.543	0.008	***	0.258	0.297	−0.039	***
Staff size: medium/large (50+ workers)	0.449	0.457	−0.008	***	0.742	0.703	0.039	***
Turnover rate (previous 12 months; prop.)	0.311	0.341	−0.030	***	0.414	0.464	−0.050	***
Vacancy rate (previous 12 months; prop.)	0.035	0.039	−0.004	***	0.060	0.069	−0.009	***
Direct care worker per service user ratio	2.426	2.203	0.223	***	1.705	1.573	0.132	***
CQC rating well-led: inadequate/requires improvement	0.233	0.264	−0.031	***	0.155	0.151	0.004	*
CQC rating well-led: good/outstanding	0.670	0.635	0.035	***	0.546	0.521	0.024	***
CQC rating well-led: no rating received	0.096	0.101	−0.004	**	0.299	0.328	−0.028	***
Unemployment rate (LA level; Office for National Statistics)	4.548	4.481	0.067	***	4.836	4.710	0.126	***
Average wage for women (LA level; Annual Survey of Hours and Earnings)	12.892	12.877	0.015	*	13.207	13.174	0.032	**
Average house price (postcode district; 2015 £)	201,245	204,904	−3,659	***	209,474	208,729	744	
Urban location	0.862	0.861	0.001		0.894	0.885	0.009	***
Unit costs residential care (LA level; £/week; 2015 £)	701.16	705.74	−4.58	***	708.24	704.03	4.21	***
Unit costs domiciliary care (LA level; £/h; 2015 £)	15.10	15.17	−0.08	***	14.88	14.86	0.02	*
Care home competition (distance-weighted Herfindahl–Hirschman Index)	0.017	0.018	−0.001	***	0.015	0.016	−0.001	***
Observations (max.)	152,247	47,143			113,123	42,642		

*Notes*: CQC = Care Quality Commission; LA = local authority.

***p < .01.

**p < .05.

*p < .1.

In terms of employer characteristics, direct care workers were, for example, relatively more likely to leave for-profit care establishments and establishments with a higher turnover and/or vacancy rate in the previous year, and establishments having a lower direct care worker per service user ratio (i.e., higher workload). On the other hand, they were less likely to leave establishments being rated by the CQC as “Good” or “Outstanding” with respect to the CQC Key Line of Enquiry “Well-led” (i.e., establishments with better management and more support for learning, innovation, and promotion of an open and fair culture; CQC, 2018).

### Estimation Results

Following previous studies, we run estimations separately by care setting. Tables 2 and 3 present the estimation results of job separation for the binary response models and a selection of covariates. The full estimation results are presented in Supplementary Tables A2 and A3. In each table, the first column includes the results from the logit estimation in the form of odds ratios. The next two columns include the marginal effects after logit and probit estimations, respectively. The results show that at the mean of the sample distribution, the estimated effects sizes of both binary response estimations are quite similar.

**Table 2.** Estimation Results of Job Separation—Residential Care (selected covariates).

Variable	(1)	(2)	(3)	(4)
	Logit	Logit	Probit	CRE Probit
	Odds Ratio	ME	ME	ME
Age	0.969***	−0.005***	−0.006***	−0.005***
	(0.003)	(0.001)	(0.001)	(0.001)
Age squared (×1,000)	1,000***	0.044***	0.052***	0.045***
	(0.040)	(0.001)	(0.001)	(0.001)
Gender: female	0.892***	−0.020***	−0.020***	−0.014***
	(0.015)	(0.003)	(0.003)	(0.003)
Distance from work (km; log)	1.183***	0.029***	0.029***	0.042***
	(0.009)	(0.001)	(0.001)	(0.011)
Qualification: yes	0.842***	−0.029***	−0.030***	−0.016**
	(0.010)	(0.002)	(0.002)	(0.008)
Training (any): yes	0.929***	−0.012***	−0.012***	0.000
	(0.011)	(0.002)	(0.002)	(0.007)
Tenure (years)	0.883***	−0.021***	−0.021***	−0.042***
	(0.003)	(0.001)	(0.001)	(0.001)
Tenure (years) squared	1.003***	0.001***	0.001***	0.001***
	(0.000)	(0.000)	(0.000)	(0.000)
Job role: care worker	0.884***	−0.021***	−0.020***	−0.001
	(0.016)	(0.003)	(0.003)	(0.008)
Job role: other care providing	0.877***	−0.023***	−0.020***	0.012
	(0.037)	(0.007)	(0.007)	(0.023)
Hourly wage (log; 2015 £)	0.429***	−0.144***	−0.132***	−0.282***
	(0.030)	(0.012)	(0.011)	(0.030)
Zero-hours contract	2.025***	0.120***	0.126***	0.125***
	(0.054)	(0.005)	(0.005)	(0.014)
Full time	0.917***	−0.015***	−0.015***	−0.026***
	(0.011)	(0.002)	(0.002)	(0.007)
Sector: for-profit	1.109***	0.018***	0.016***	0.009*
	(0.037)	(0.006)	(0.005)	(0.005)
Sector: not-for-profit	0.916**	−0.014**	−0.015***	−0.020***
	(0.032)	(0.006)	(0.006)	(0.005)
Staff size: medium/large (50+ workers)	0.941***	−0.010***	−0.012***	−0.012*
	(0.012)	(0.002)	(0.002)	(0.007)
Turnover rate (previous 12 months)	1.136***	0.022***	0.022***	−0.006
	(0.018)	(0.003)	(0.003)	(0.006)
Vacancy rate (previous 12 months)	1.242***	0.037***	0.037***	0.021
	(0.098)	(0.013)	(0.014)	(0.025)
CQC rating (well-led): good/outstanding	0.869***	−0.024***	−0.025***	−0.019***
	(0.012)	(0.002)	(0.002)	(0.004)
CQC rating (well-led): not rated	0.859***	−0.026***	−0.026***	−0.013**
	(0.021)	(0.004)	(0.004)	(0.006)

*Notes*: CRE = conditional random effect; CQC = Care Quality Commission; LA = local authority; ME = marginal effect. Robust standard errors in parentheses. Base categories: qualification: no qualification; training: no training received; job role: senior care worker; sector: statutory LA; staff size: micro/small (1−49 workers); CQC rating: inadequate/requires improvement. Supplementary Table A2 presents the estimation results for the full set of covariates as well as the statistics associated with each estimation.

***p < .01.

**p < .05.

*p < .1.

**Table 3.** Estimation Results of Job Separation—Domiciliary Care (selected covariates).

Variable	(1)	(2)	(3)	(4)
	Logit	Logit	Probit	CRE Probit
	Odds ratio	ME	ME	ME
Age	0.952***	−0.009***	−0.010***	−0.008***
	(0.003)	(0.001)	(0.001)	(0.001)
Age squared (×1,000)	1,000***	0.081***	0.087***	0.065***
	(0.041)	(0.001)	(0.001)	(0.001)
Gender: female	0.982	−0.003	−0.004	−0.001
	(0.018)	(0.004)	(0.004)	(0.003)
Distance from work (km; log)	1.155***	0.027***	0.027***	−0.002
	(0.009)	(0.001)	(0.001)	(0.010)
Qualification: yes	0.957***	−0.008***	−0.009***	−0.016*
	(0.012)	(0.002)	(0.002)	(0.008)
Training (any): yes	1.049***	0.009***	0.009***	0.018**
	(0.014)	(0.003)	(0.003)	(0.008)
Tenure (years)	0.879***	−0.024***	−0.024***	−0.052***
	(0.003)	(0.001)	(0.001)	(0.001)
Tenure (years) squared	1.003***	0.001***	0.001***	0.002***
	(0.000)	(0.000)	(0.000)	(0.000)
Job role: care worker	1.114***	0.020***	0.020***	0.032**
	(0.033)	(0.005)	(0.005)	(0.013)
Job role: other care providing	1.079**	0.014**	0.014**	0.044**
	(0.041)	(0.007)	(0.007)	(0.021)
Hourly wage (log; 2015 £)	0.667***	−0.077***	−0.075***	−0.277***
	(0.044)	(0.012)	(0.012)	(0.031)
Zero-hours contract	1.069***	0.013***	0.013***	0.030**
	(0.016)	(0.003)	(0.003)	(0.012)
Full time	0.924***	−0.015***	−0.015***	−0.043***
	(0.011)	(0.002)	(0.002)	(0.010)
Sector: for-profit	0.824***	−0.038***	−0.037***	−0.042***
	(0.025)	(0.006)	(0.006)	(0.006)
Sector: not-for-profit	0.704***	−0.067***	−0.065***	−0.064***
	(0.024)	(0.007)	(0.006)	(0.006)
Staff size: medium/large (50+ workers)	0.901***	−0.020***	−0.020***	−0.043***
	(0.013)	(0.003)	(0.003)	(0.008)
Turnover rate (previous 12 months)	1.175***	0.031***	0.030***	−0.011**
	(0.015)	(0.002)	(0.002)	(0.004)
Vacancy rate (previous 12 months)	1.262***	0.044***	0.044***	0.027
	(0.071)	(0.011)	(0.011)	(0.024)
CQC rating (well-led): good/outstanding	0.956***	−0.009***	−0.009***	−0.007
	(0.016)	(0.003)	(0.003)	(0.005)
CQC rating (well-led): not rated	1.093***	0.017***	0.018***	0.004
	(0.023)	(0.004)	(0.004)	(0.006)

*Notes*: CRE = conditional random effect; CQC = Care Quality Commission; LA = local authority; ME = marginal effect. Robust standard errors in parentheses. base categories: qualification: no qualification; training: no training received; job role: senior care worker; sector: statutory LA; staff size: micro/small (1−49 workers); CQC rating: inadequate/requires improvement. Supplementary Table A3 presents the estimation results for the full set of covariates as well as the statistics associated with each estimation.

***p < .01.

**p < .05.

*p < .1.

The results from the estimation controlling for unobserved heterogeneity (i.e., CRE probit) are reported in column 4. The *F*-test of joint statistical significance of ${\bar{z}}_{ij}$ in CRE probit ($\chi^{2}$ value of 30,378 [*p* value of <.001] for residential care and 25,909 [*p* value of <.001] for domiciliary care) confirms that unobserved heterogeneity significantly affects estimation results. Therefore, the *within* CRE probit estimation results are to be preferred over those from simple pooled probit.

When controlling for unobserved heterogeneity (i.e., CRE probit estimation), the wage effect is about twice as large for residential direct care workers (i.e., −0.28 in the CRE probit compared to −0.13 in the pooled probit estimation) and about three times larger for domiciliary direct care workers (i.e., −0.28 in the CRE probit compared to −0.08 in the pooled probit estimation). These results suggest that, as expected, not accounting for unobserved effects led to a substantial downward bias in the wage effect on job separation.

**Figure 1.**
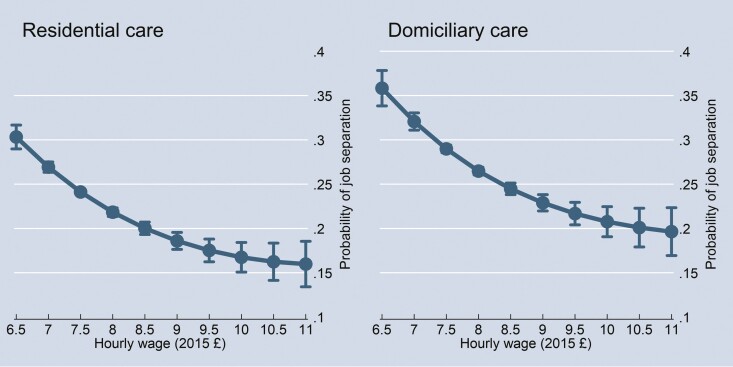
Predicted probabilities of job separation by hourly wage. *Note*: Based on CRE probit estimations with three-degree wage polynomial. CRE = conditional random effect.

We explored the functional form of the relationship between wages and job separation by estimating a model with a three-degree wage polynomial. The predicted job separation rates by wage (Figure 1) revealed that the wage effect had diminishing marginal magnitudes, that is, an increase in wage had a stronger effect on reducing job separations at lower wage levels and a weaker effect at higher wage levels.

We found evidence of omitted variable bias toward zero for other job characteristics as well. The only exception was employment on contracts without guaranteed hours (i.e., zero-hours contracts) in the case of residential care, which was strongly related (12.5 ppt higher probability) to leaving the employer both in the pooled probit and CRE probit estimations. For domiciliary care, the effect of employment on zero-hours contracts on the likelihood of job separation more than doubled when controlling for unobserved heterogeneity (i.e., from 1.3 ppt in pooled probit to 3 ppt in CRE probit). The effect of full-time employment on the probability of leaving direct care job increased to −2.6 ppt in the CRE probit estimation for residential care (from −1.5 ppt in the pooled probity estimation) and to −4.3 ppt in the CRE probit estimation for domiciliary care (from −1.5 ppt in the pooled probit estimation).

After controlling for unobserved heterogeneity, the effect of having received training on the probability of job separation turned for residential care direct care workers from small negative (−1.2 ppt in pooled probit) to zero, while for domiciliary care direct care workers from small positive (0.9 ppt in pooled probit) to larger positive (1.8 ppt in CRE probit). The absence or even a positive relationship between training incidence and job separation of direct care workers in England could be related to the lack of pay progression in LTC and, therefore, low rewards to additionally acquired skills.

In terms of employer characteristics, we found that care homes with better management (i.e., rated as “Good” or “Outstanding”) had a 1.9 ppt higher probability to retain direct care workers, confirming that good management is important for increasing direct care workers’ retention. We found no similar effect for domiciliary care employers. On the other hand, larger domiciliary care establishments were significantly less likely to lose direct care staff (−4.3 ppt) compared to small and medium ones. We also found that not-for-profit care establishments were slightly better at retaining direct care workers compared to public and for-profit establishments (i.e., 1–2 ppt lower probability of job separation).

Despite a significant positive relationship between the probability of job separation and the employer-level turnover and vacancy rates in the previous year in the logit and probit estimations, once we controlled for unobserved heterogeneity the coefficients turned small and statistically insignificant. The only exception was the effect of employer-level turnover rate in a domiciliary care setting, which was small negative (−1.1 ppt). This would rather show that both job separations and the overall employer turnover and vacancy rates might have been related to some unobserved employer-level characteristics, whose effects were removed by the CRE probit estimation.

## Discussion and Implications

Since the 1990s England’s councils with LTC responsibilities have started to reduce the amount of LTC services they provide directly, and nowadays, about 9 in 10 care staff are employed by independent (i.e., for-profit and not-for-profit) providers. The outsourcing of LTC services has provided financial savings to the public purse, but at the cost of the LTC workforce. Direct care workers employed by the independent sector (in particular, for-profit providers) have low wages (on average 35% are paid just the statutory minimum wage), a high share is employed on contracts without guaranteed hours (e.g., 56% of domiciliary direct care workers), and the majority are paid only the substantially lower statutory sick pay (Skills for Care, 2020b, 2022). A report by Community Integrated Care estimated that the pay gap between direct care workers employed in the independent sector and those employed in equivalent public sector roles in local authorities and the National Health Service (NHS) is about 40% or nearly £7,000 per year (Community Integrated Care, 2021).

Considering the above, it is not surprising that staff turnover in the independent LTC sector in England is relatively high (34%) compared to the public sector LTC and equivalent care roles in the NHS (i.e., healthcare assistants; 13%), and that a large share of direct care workers is using LTC jobs as a stepping stone to better jobs in the NHS (Kelly et al., 2022; Skills for Care, 2020b). Finding practical solutions to the persistent staff turnover in LTC is an important issue faced by care providers and policy-makers. Its importance is linked to the sustainability of a care workforce able to provide good-quality services (Allan and Vadean, 2021; Castle et al., 2007; Eastwood, 2017; Netten et al., 2007; Skills for Care, 2020b) as well as the need to increase future LTC supply in line with the predicted increase in LTC demand (Atkins et al., 2019; National Audit Office, 2018; Wittenberg et al., 2019).

This study focused on assessing the relationship between job quality (e.g., wages and guaranteed working hours) and separation from the employer of direct care workers in LTC. Using longitudinal data for England from the Adult Social Care Workforce Data Set (ASC-WDS), we found a statistically significant negative effect of wages on actual job separation and a downward bias of the wage effect (i.e., closer to zero) when not controlling for (time-invariant) unobserved heterogeneity. The size of the omitted variable bias correction is larger than found in previous studies accounting for the endogeneity of wages. Nonetheless, it is not directly comparable, as, for example, Baughman and Smith (2012) looked at transitions out of a direct care occupation, whereas Powers and Powers (2010) analyzed wage effects on employer-level turnover rates.

The wage effect is not very large, but not unimportant, and larger at lower wage levels (i.e., diminishing marginal effect). Translated to the differences between sectors mentioned above, a 26% hourly wage increase from the sample mean of direct care workers employed by independent residential care establishments (£7.59 in 2015 £) to the sample mean of direct care workers employed by public residential care establishments (£9.60 in 2015 £) would reduce the probability of job separation by 27%. Similarly, a 23% hourly wage increase from the sample mean of direct care workers employed by independent domiciliary care establishments (£7.80 in 2015 £) to the sample mean of direct care workers employed by public domiciliary care establishments (£9.60 in 2015 £) would reduce the probability of job separation by 22%.

The identified wage effect on job separations of direct care workers in LTC is larger than that found for registered nurses employed by the NHS in England: A 10% hourly wage increase was found to reduce the probability of nurses leaving each year by about 6% (Frijters et al., 2007). The lower responsiveness of registered nurses to changes in wages is probably related to the significant power the NHS has on the labor market for nurses in England and the limited alternative job opportunities.

Increasing wages for LTC staff in the independent sector could be combined with other aspects of job quality for a more meaningful reduction in direct care worker turnover. One such aspect is employment contract type, with our results confirming that employment on full-time contracts and contracts with guaranteed working hours both have a positive effect on staff retention. While contracts without guaranteed working hours may give employers more flexibility to adapt to fluctuations in demand, they might represent a false economy, as they lead to increased staff turnover and, therefore, higher recruitment and induction costs.

In contrast to previous studies that found training provision to improve job satisfaction and reduce turnover of nurse aids in nursing homes (Castle et al., 2007), we found that training is not improving retention of DWC working in care homes, and is even likely to slightly increase job separation of domiciliary frontline staff (+1.8 ppt). This is not surprising considering that the current pay structure in LTC in England allows little progression for skills and experience (Skills for Care, 2020b). A concern for the LTC sector would be that this may create disincentives for care providers to invest in staff skills, with long-term consequences for the quality of services (Moriarty et al., 2018).

Our results confirm previous findings that good management reduces direct care workers’ turnover. This could be related to management styles giving DWCs more job autonomy and/or allowing them to contribute to care planning (Donoghue and Castle, 2009). However, the effect is small and statistically significant only for the care home setting. This finding aligns, though, with that from Towers et al. (2021), showing that good management is not only improving staff retention, but also care home residents’ quality of life.

To conclude, we find that improving pay and employment conditions for direct care workers would reduce staff turnover in England’s LTC sector. A coherent strategy could be to align pay and conditions in the independent sector to those in the public sector. This will come, however, at a cost to the local and central governments, as tariffs paid by local councils will need to increase for care providers to be able to afford to pay higher wages, improve pay progression, and offer full-time contracts with guaranteed hours.

### Limitations

Omitted variable bias was addressed in this study by using panel data econometric methods accounting for time-invariant unobserved factors (i.e., *within* estimators). Nonetheless, our results may still be biased by time-variant unobserved factors at worker or employer level. We believe this bias to be small as the data set allowed the inclusion of a large number of controls on worker, job, employer, and local market characteristics, shown to be important confounders in previous studies.

With respect to wages, taking into account both time-variant and time-invariant unobserved factors—by identifying valid instruments and using Instrumental Variables FE estimators—would likely lead to the identification of an even stronger (i.e., further from zero) wage effect on job separations. We could, therefore, consider the wage effect found in this study as a lower bound.

## Supplementary Material

igad009_suppl_Supplementary_Material

## Data Availability

The data used for this analysis were obtained from Skills for Care under a Data Sharing Agreement. The code used for the analysis is available from the authors upon request.
