# Supplementary material for: Job Quality and Job Separation of Direct Care Workers in England
Source: Innov Aging. 2023 Jan 31;7(2):igad009. doi: 10.1093/geroni/igad009 (PMC10024478; doi:10.1093/geroni/igad009)
Supplement: igad009_suppl_Supplementary_Material [file igad009_suppl_supplementary_material.docx]

*Innovation in Aging* Online Supplementary Material: Florin Vadean & Eirini-Christina Saloniki. Job Quality and Job Separation of Direct Care Workers in England.

**Table A1.** Sample representativeness – establishment level

| Variable | Residential care | | | | | | Domiciliary care | | | | | |
| --- | --- | --- | --- | --- | --- | --- | --- | --- | --- | --- | --- | --- |
|  | CQC directory | | | ASC-WDS sample | | | CQC directory | | | ASC-WDS sample | | |
|  | 2016 | 2017 | 2018 | 2016 | 2017 | 2018 | 2016 | 2017 | 2018 | 2016 | 2017 | 2018 |
| Sector: statutory LA | 0.03 | 0.03 | 0.03 | 0.05 | 0.05 | 0.06 | 0.04 | 0.03 | 0.03 | 0.07 | 0.07 | 0.09 |
| Sector: for-profit | 0.81 | 0.82 | 0.82 | 0.77 | 0.78 | 0.80 | 0.83 | 0.85 | 0.86 | 0.80 | 0.81 | 0.81 |
| Sector: not-for-profit | 0.16 | 0.15 | 0.15 | 0.19 | 0.17 | 0.15 | 0.13 | 0.12 | 0.11 | 0.13 | 0.12 | 0.11 |
| Service type: care home w/ nursing | 0.27 | 0.27 | 0.28 | 0.28 | 0.29 | 0.33 |  |  |  |  |  |  |
| Service type: care home w/o nursing | 0.73 | 0.73 | 0.72 | 0.72 | 0.71 | 0.67 |  |  |  |  |  |  |
| Capacity (i.e., care home beds) | 27.84 | 28.30 | 28.72 | 33.23 | 34.25 | 35.51 |  |  |  |  |  |  |
| CQC rating: no rating received | 0.20 | 0.08 | 0.06 | 0.15 | 0.05 | 0.04 | 0.53 | 0.30 | 0.25 | 0.36 | 0.13 | 0.09 |
| CQC rating: Inadequate/Req. improvement | 0.23 | 0.20 | 0.18 | 0.20 | 0.17 | 0.16 | 0.10 | 0.12 | 0.11 | 0.12 | 0.13 | 0.09 |
| CQC rating: Good/Outstanding | 0.57 | 0.73 | 0.76 | 0.65 | 0.79 | 0.80 | 0.37 | 0.58 | 0.64 | 0.52 | 0.74 | 0.82 |
| Region: East | 0.11 | 0.11 | 0.11 | 0.13 | 0.13 | 0.13 | 0.12 | 0.12 | 0.12 | 0.12 | 0.12 | 0.11 |
| Region: East Midlands | 0.10 | 0.10 | 0.10 | 0.09 | 0.10 | 0.09 | 0.09 | 0.09 | 0.10 | 0.07 | 0.07 | 0.07 |
| Region: London | 0.09 | 0.09 | 0.09 | 0.06 | 0.05 | 0.04 | 0.14 | 0.15 | 0.16 | 0.10 | 0.10 | 0.10 |
| Region: North East | 0.05 | 0.05 | 0.05 | 0.06 | 0.06 | 0.06 | 0.04 | 0.03 | 0.03 | 0.05 | 0.06 | 0.05 |
| Region: North West | 0.12 | 0.12 | 0.12 | 0.12 | 0.12 | 0.11 | 0.12 | 0.12 | 0.11 | 0.14 | 0.15 | 0.14 |
| Region: South East | 0.19 | 0.19 | 0.19 | 0.17 | 0.17 | 0.17 | 0.16 | 0.16 | 0.16 | 0.17 | 0.16 | 0.17 |
| Region: South West | 0.14 | 0.13 | 0.13 | 0.13 | 0.13 | 0.15 | 0.11 | 0.10 | 0.10 | 0.12 | 0.11 | 0.12 |
| Region: West Midlands | 0.11 | 0.11 | 0.11 | 0.12 | 0.12 | 0.13 | 0.13 | 0.13 | 0.13 | 0.12 | 0.12 | 0.12 |
| Region: Yorkshire and the Humber | 0.10 | 0.10 | 0.09 | 0.12 | 0.13 | 0.13 | 0.09 | 0.09 | 0.09 | 0.10 | 0.11 | 0.11 |
| Observations | 16,385 | 16,073 | 15,780 | 4,635 | 4,295 | 3,653 | 8,100 | 8,369 | 8,800 | 1,757 | 1,750 | 1,567 |

Source: Care Quality Commission care directory; Adult Social Care Workforce Data Set (ASC-WDS).

**Table A2.** Estimation results of job separation – residential care

| Variable | (1) | (2) | (3) | (4) | (5) | (6) |
| --- | --- | --- | --- | --- | --- | --- |
|  | logit | logit | probit | LPM/OLS | CRE probit | FE LPM |
|  | Odds ratios | ME | ME | $\beta$ | ME | $\beta$ |
| Age | 0.969*** | -0.005*** | -0.006*** | -0.008*** | -0.005*** |  |
|  | (0.003) | (0.001) | (0.001) | (0.001) | (0.001) |  |
| Age squared (x 1,000) | 1,000*** | 0.044*** | 0.052*** | 0.069*** | 0.045*** |  |
|  | (0.040) | (0.001) | (0.001) | (0.001) | (0.001) |  |
| Gender: female | 0.892*** | -0.020*** | -0.020*** | -0.020*** | -0.014*** |  |
|  | (0.015) | (0.003) | (0.003) | (0.003) | (0.003) |  |
| Nationality: British | 0.953*** | -0.008*** | -0.009*** | -0.009*** | -0.034 | -0.056** |
|  | (0.015) | (0.003) | (0.003) | (0.003) | (0.032) | (0.029) |
| Distance from work (km; log) | 1.183*** | 0.029*** | 0.029*** | 0.030*** | 0.042*** | 0.054*** |
|  | (0.009) | (0.001) | (0.001) | (0.001) | (0.011) | (0.012) |
| Qualification: yes | 0.842*** | -0.029*** | -0.030*** | -0.034*** | -0.016** | 0.017** |
|  | (0.010) | (0.002) | (0.002) | (0.002) | (0.008) | (0.008) |
| Training (any): yes | 0.929*** | -0.012*** | -0.012*** | -0.014*** | 0.000 | 0.019*** |
|  | (0.011) | (0.002) | (0.002) | (0.002) | (0.007) | (0.007) |
| Tenure (years) | 0.883*** | -0.021*** | -0.021*** | -0.020*** | -0.042*** |  |
|  | (0.003) | (0.001) | (0.001) | (0.000) | (0.001) |  |
| Tenure (years) squared | 1.003*** | 0.001*** | 0.001*** | 0.001*** | 0.001*** |  |
|  | (0.000) | (0.000) | (0.000) | (0.000) | (0.000) |  |
| Job role: care worker | 0.884*** | -0.021*** | -0.020*** | -0.018*** | -0.001 | -0.014* |
|  | (0.016) | (0.003) | (0.003) | (0.003) | (0.008) | (0.008) |
| Job role: other care-providing | 0.877*** | -0.023*** | -0.020*** | -0.020*** | 0.012 | -0.007 |
|  | (0.037) | (0.007) | (0.007) | (0.007) | (0.023) | (0.020) |
| Hourly wage (log; 2015 £) | 0.429*** | -0.144*** | -0.132*** | -0.122*** | -0.282*** | -0.207*** |
|  | (0.030) | (0.012) | (0.011) | (0.010) | (0.030) | (0.027) |
| Zero-hours contract | 2.025*** | 0.120*** | 0.126*** | 0.155*** | 0.125*** | 0.179*** |
|  | (0.054) | (0.005) | (0.005) | (0.006) | (0.014) | (0.017) |
| Full-time | 0.917*** | -0.015*** | -0.015*** | -0.015*** | -0.026*** | -0.026*** |
|  | (0.011) | (0.002) | (0.002) | (0.002) | (0.007) | (0.007) |
| Sector: for-profit | 1.109*** | 0.018*** | 0.016*** | 0.014*** | 0.009* |  |
|  | (0.037) | (0.006) | (0.005) | (0.005) | (0.005) |  |
| Sector: not-for-profit | 0.916** | -0.014** | -0.015*** | -0.018*** | -0.020*** |  |
|  | (0.032) | (0.006) | (0.006) | (0.005) | (0.005) |  |
| Care setting: CH w/o nursing | 1.011 | 0.002 | 0.001 | 0.001 | -0.003 |  |
|  | (0.014) | (0.002) | (0.002) | (0.002) | (0.002) |  |
| User type: young adults | 0.975* | -0.004* | -0.003 | -0.005** | -0.001 | -0.016 |
|  | (0.015) | (0.003) | (0.003) | (0.003) | (0.040) | (0.036) |
| User type: mixed | 0.964*** | -0.006*** | -0.006** | -0.007*** | 0.003 | 0.006 |
|  | (0.013) | (0.002) | (0.002) | (0.002) | (0.019) | (0.018) |
| Staff size: medium/large (50+ workers) | 0.941*** | -0.010*** | -0.012*** | -0.010*** | -0.012* | -0.011* |
|  | (0.012) | (0.002) | (0.002) | (0.002) | (0.007) | (0.006) |
| Turnover rate (previous 12 months) | 1.136*** | 0.022*** | 0.022*** | 0.023*** | -0.006 | -0.004 |
|  | (0.018) | (0.003) | (0.003) | (0.003) | (0.006) | (0.006) |
| Vacancy rate (previous 12 months) | 1.242*** | 0.037*** | 0.037*** | 0.041*** | 0.021 | 0.049** |
|  | (0.098) | (0.013) | (0.014) | (0.014) | (0.025) | (0.024) |
| Direct care worker per service user ratio | 0.995*** | -0.001*** | -0.001*** | -0.001*** | -0.000 | -0.000 |
|  | (0.001) | (0.000) | (0.000) | (0.000) | (0.001) | (0.001) |
| CQC rating (Well-led): Good/Outstanding | 0.869*** | -0.024*** | -0.025*** | -0.025*** | -0.019*** | -0.018*** |
|  | (0.012) | (0.002) | (0.002) | (0.002) | (0.004) | (0.004) |
| CQC rating (Well-led): Not rated | 0.859*** | -0.026*** | -0.026*** | -0.027*** | -0.013** | -0.015*** |
|  | (0.021) | (0.004) | (0.004) | (0.004) | (0.006) | (0.005) |
| Unemployment rate (LA level) | 1.013** | 0.002** | 0.002** | 0.002** | 0.001 | 0.006*** |
|  | (0.006) | (0.001) | (0.001) | (0.001) | (0.002) | (0.002) |
| Average wage for women (LA level; log; 2015 £) | 0.972 | -0.005 | -0.007 | -0.005 | -0.036 | -0.052* |
|  | (0.066) | (0.012) | (0.012) | (0.012) | (0.031) | (0.029) |
| House price (Postcode district level; log; 2015 £) | 1.070*** | 0.011*** | 0.011*** | 0.010*** | 0.015 | 0.048 |
|  | (0.024) | (0.004) | (0.004) | (0.004) | (0.033) | (0.031) |
| Urban | 1.061*** | 0.010*** | 0.011*** | 0.010*** | 0.007*** |  |
|  | (0.017) | (0.003) | (0.003) | (0.003) | (0.003) |  |
| Unit Costs Residential Care (LA level; log; £/week) | 1.217*** | 0.033*** | 0.033*** | 0.033*** | -0.063*** | -0.034** |
|  | (0.057) | (0.008) | (0.008) | (0.008) | (0.018) | (0.017) |
| Unit Costs Domiciliary Care (LA level; log; £/hour) | 1.099 | 0.016 | 0.016 | 0.016 | 0.035** | 0.043*** |
|  | (0.068) | (0.011) | (0.011) | (0.011) | (0.016) | (0.015) |
| Care home competition (distance-weighted HHI) | 1.671 | 0.088 | 0.094 | 0.081 | 2.965** | 1.677 |
|  | (0.640) | (0.065) | (0.065) | (0.066) | (1.246) | (1.083) |
| Year | yes | yes | yes | yes | yes | yes |
| Region | yes | yes | yes | yes | yes |  |
| Constant | 0.813 |  |  | 0.432*** |  | 0.116 |
|  | (0.321) |  |  | (0.066) |  | (0.403) |
| Observations | 199,390 | 199,390 | 199,390 | 199,390 | 199,390 | 199,390 |
| No. of job spells |  |  |  |  |  | 118,503 |
| R-sq; Pseudo R-sq | 0.051 | 0.051 | 0.051 | 0.054 | 0.199 | 0.180 |
| Log likelihood/pseudo-likelihood | -103,481 | -103,481 | -103,514 |  | -87,322 |  |
| Hausman test FE vs. RE (chi-sq)/ F-test of $\bar{z}_{ij}$=0 |  |  |  |  | 30,378*** | 24,189*** |
| Sigma ui |  |  |  |  |  | 0.471 |
| Sigma eij |  |  |  |  |  | 0.292 |
| Rho |  |  |  |  |  | 0.722 |

*Notes:* Robust standard errors in parentheses. Base categories: Nationality: Other; Qualification: no qualification; Training: no training received; Job role: senior care worker; Sector: statutory LA; User type: old age; Care setting: care home with nursing; Staff size: micro/small (1-49 workers); CQC rating: Inadequate/Requires improvement. LPM: linear probability model; CRE: conditional random effects; FE: fixed effects. Significance level: *** p<0.01, ** p<0.05, * p<0.1

**Table A3.** Estimation results of job separation – domiciliary care

| Variable | (1) | (2) | (3) | (4) | (5) | (6) |
| --- | --- | --- | --- | --- | --- | --- |
|  | logit | logit | probit | LPM/OLS | CRE probit | FE LPM |
|  | Odds ratios | ME | ME | $\beta$ | ME | $\beta$ |
| Age | 0.952*** | -0.009*** | -0.010*** | -0.011*** | -0.008*** |  |
|  | (0.003) | (0.001) | (0.001) | (0.001) | (0.001) |  |
| Age squared (x 1,000) | 1,000*** | 0.081*** | 0.087*** | 0.103*** | 0.065*** |  |
|  | (0.041) | (0.001) | (0.001) | (0.001) | (0.001) |  |
| Gender: female | 0.982 | -0.003 | -0.004 | -0.003 | -0.001 |  |
|  | (0.018) | (0.004) | (0.004) | (0.003) | (0.003) |  |
| Nationality: British | 0.900*** | -0.020*** | -0.020*** | -0.021*** | -0.018 | -0.017 |
|  | (0.017) | (0.004) | (0.004) | (0.004) | (0.019) | (0.017) |
| Distance from work (km; log) | 1.155*** | 0.027*** | 0.027*** | 0.027*** | -0.002 | -0.003 |
|  | (0.009) | (0.001) | (0.001) | (0.001) | (0.010) | (0.010) |
| Qualification: yes | 0.957*** | -0.008*** | -0.009*** | -0.010*** | -0.016* | 0.004 |
|  | (0.012) | (0.002) | (0.002) | (0.003) | (0.008) | (0.008) |
| Training (any): yes | 1.049*** | 0.009*** | 0.009*** | 0.009*** | 0.018** | 0.033*** |
|  | (0.014) | (0.003) | (0.003) | (0.003) | (0.008) | (0.008) |
| Tenure (years) | 0.879*** | -0.024*** | -0.024*** | -0.023*** | -0.052*** |  |
|  | (0.003) | (0.001) | (0.001) | (0.001) | (0.001) |  |
| Tenure (years) squared | 1.003*** | 0.001*** | 0.001*** | 0.001*** | 0.002*** |  |
|  | (0.000) | (0.000) | (0.000) | (0.000) | (0.000) |  |
| Job role: care worker | 1.114*** | 0.020*** | 0.020*** | 0.018*** | 0.032** | 0.021 |
|  | (0.033) | (0.005) | (0.005) | (0.005) | (0.013) | (0.013) |
| Job role: other care-providing | 1.079** | 0.014** | 0.014** | 0.013** | 0.044** | 0.026 |
|  | (0.041) | (0.007) | (0.007) | (0.006) | (0.021) | (0.019) |
| Hourly wage (log; 2015 £) | 0.667*** | -0.077*** | -0.075*** | -0.067*** | -0.277*** | -0.229*** |
|  | (0.044) | (0.012) | (0.012) | (0.011) | (0.031) | (0.028) |
| Zero-hours contract | 1.069*** | 0.013*** | 0.013*** | 0.013*** | 0.030** | 0.029** |
|  | (0.016) | (0.003) | (0.003) | (0.003) | (0.012) | (0.012) |
| Full-time | 0.924*** | -0.015*** | -0.015*** | -0.015*** | -0.043*** | -0.047*** |
|  | (0.011) | (0.002) | (0.002) | (0.002) | (0.010) | (0.010) |
| Sector: for-profit | 0.824*** | -0.038*** | -0.037*** | -0.037*** | -0.042*** |  |
|  | (0.025) | (0.006) | (0.006) | (0.005) | (0.006) |  |
| Sector: not-for-profit | 0.704*** | -0.067*** | -0.065*** | -0.064*** | -0.064*** |  |
|  | (0.024) | (0.007) | (0.006) | (0.006) | (0.006) |  |
| User type: young adults | 0.865*** | -0.026*** | -0.026*** | -0.025*** | 0.242*** | 0.307*** |
|  | (0.027) | (0.006) | (0.006) | (0.006) | (0.064) | (0.072) |
| User type: mixed | 1.044* | 0.008* | 0.008* | 0.009* | 0.106*** | 0.169*** |
|  | (0.026) | (0.005) | (0.005) | (0.005) | (0.035) | (0.048) |
| Staff size: medium/large (50+ workers) | 0.901*** | -0.020*** | -0.020*** | -0.021*** | -0.043*** | -0.032*** |
|  | (0.013) | (0.003) | (0.003) | (0.003) | (0.008) | (0.007) |
| Turnover rate (previous 12 months) | 1.175*** | 0.031*** | 0.030*** | 0.033*** | -0.011** | 0.004 |
|  | (0.015) | (0.002) | (0.002) | (0.003) | (0.004) | (0.004) |
| Vacancy rate (previous 12 months) | 1.262*** | 0.044*** | 0.044*** | 0.046*** | 0.027 | 0.007 |
|  | (0.071) | (0.011) | (0.011) | (0.011) | (0.024) | (0.024) |
| Direct care worker per service user ratio | 1.004*** | 0.001*** | 0.001*** | 0.001*** | -0.001 | -0.001 |
|  | (0.001) | (0.000) | (0.000) | (0.000) | (0.001) | (0.001) |
| CQC rating (Well-led): Good/Outstanding | 0.956*** | -0.009*** | -0.009*** | -0.008** | -0.007 | -0.006 |
|  | (0.016) | (0.003) | (0.003) | (0.003) | (0.005) | (0.005) |
| CQC rating (Well-led): Not rated | 1.093*** | 0.017*** | 0.018*** | 0.019*** | 0.004 | -0.007 |
|  | (0.023) | (0.004) | (0.004) | (0.004) | (0.006) | (0.005) |
| Unemployment rate (LA level) | 0.954*** | -0.009*** | -0.009*** | -0.009*** | 0.005** | 0.009*** |
|  | (0.006) | (0.001) | (0.001) | (0.001) | (0.003) | (0.002) |
| Average wage for women (LA level; log; 2015 £) | 1.909*** | 0.122*** | 0.122*** | 0.123*** | -0.125*** | -0.078** |
|  | (0.129) | (0.013) | (0.013) | (0.013) | (0.036) | (0.035) |
| House price (Postcode district level; log; 2015 £) | 1.066*** | 0.012*** | 0.012*** | 0.011*** | 0.055** | 0.054** |
|  | (0.025) | (0.004) | (0.004) | (0.004) | (0.022) | (0.021) |
| Urban | 1.041** | 0.008** | 0.008** | 0.007* | 0.006 |  |
|  | (0.021) | (0.004) | (0.004) | (0.004) | (0.004) |  |
| Unit Costs Residential Care (LA level; log; £/week) | 1.221*** | 0.038*** | 0.037*** | 0.038*** | -0.029 | 0.002 |
|  | (0.064) | (0.010) | (0.010) | (0.010) | (0.020) | (0.020) |
| Unit Costs Domiciliary Care (LA level; log; £/hour) | 0.707*** | -0.065*** | -0.064*** | -0.063*** | -0.098*** | -0.104*** |
|  | (0.048) | (0.013) | (0.013) | (0.013) | (0.019) | (0.019) |
| Care home competition (distance-weighted HHI) | 3.329*** | 0.227*** | 0.228*** | 0.241*** | -0.578 | 0.565 |
|  | (1.197) | (0.068) | (0.069) | (0.073) | (0.639) | (0.717) |
| Year | yes | yes | yes | yes | yes | yes |
| Region | yes | yes | yes | yes | yes |  |
| Constant | 0.319*** |  |  | 0.276*** |  | 0.158 |
|  | (0.136) |  |  | (0.080) |  | (0.329) |
| Observations | 155,765 | 155,765 | 155,765 | 155,765 | 155,765 | 155,765 |
| No. of job spells |  |  |  |  |  | 92,780 |
| R-sq; Pseudo R-sq | 0.042 | 0.042 | 0.042 | 0.048 | 0.204 | 0.208 |
| Log likelihood/pseudo-likelihood | -87,586 | -87,586 | -87,609 |  | -72,818 |  |
| Hausman test FE vs. RE (chi-sq)/ F-test of $\bar{z}_{ij}$=0 |  |  |  |  | 25,909*** | 22,490*** |
| Sigma ui |  |  |  |  |  | 0.501 |
| Sigma eij |  |  |  |  |  | 0.309 |
| Rho |  |  |  |  |  | 0.724 |

Notes: Robust standard errors in parentheses. Base categories: Nationality: Other; Qualification: no qualification; Training: no training received; Job role: senior care worker; Sector: statutory LA; User type: old age; Staff size: micro/small (1-49 workers); CQC rating: Inadequate/Requires improvement. LPM: linear probability model; CRE: conditional random effects; FE: fixed effects. Significance level: *** p<0.01, ** p<0.05, * p<0.1
